# Brain Metabolite, N-Acetylaspartate Is a Potent Protein Aggregation Inhibitor

**DOI:** 10.3389/fncel.2021.617308

**Published:** 2021-02-05

**Authors:** Marina Warepam, Awdhesh Kumar Mishra, Gurumayum Suraj Sharma, Kritika Kumari, Snigdha Krishna, Mohd Sajjad Ahmad Khan, Hamidur Rahman, Laishram Rajendrakumar Singh

**Affiliations:** ^1^Department of Biotechnology, Manipur University, Manipur, India; ^2^Department of Biotechnology, Yeungnam University, Gyeongsan-si, South Korea; ^3^Department of Botany, Bhaskaracharya College of Applied Sciences, University of Delhi, New Delhi, India; ^4^Dr. B. R. Ambedkar Center for Biomedical Research, University of Delhi, New Delhi, India; ^5^Department of Basic Sciences, Deanship of Preparatory Year and Supporting Studies, Imam Abdulrahman Bin Faisal University, Dammam, Saudi Arabia

**Keywords:** protein aggregation, light scattering, protein stability, protein denaturation, neurological disorders

## Abstract

Deposition of toxic protein inclusions is a common hallmark of many neurodegenerative disorders including Alzheimer's disease, Parkinson disease etc. N-acetylaspartate (NAA) is an important brain metabolite whose levels got altered under various neurodegenerative conditions. Indeed, NAA has been a widely accepted biological marker for various neurological disorders. We have also reported that NAA is a protein stabilizer. In the present communication, we investigated the role of NAA in modulating the aggregation propensity on two model proteins (carbonic anhydrase and catalase). We discovered that NAA suppresses protein aggregation and could solubilize preformed aggregates.

## Introduction

The amino acid, N-acetylaspartate (NAA) is synthesized in the brain from aspartate and acetyl-coenzyme A by the enzyme L-aspartate N-acetyltransferase via acetylation of aspartate by acetyl coenzyme A. On the other hand, it is also catabolized by the enzyme, aspartoacylase (Birnbaum et al., 1952; Goldstein, 1959; Knizley, 1967; Truckenmiller et al., 1985). NAA is found at exceptionally high concentrations in various regions of the brain and can attain up to 10 mM or more (Miyake et al., 1981; Blüml, 1999; Pan and Takahashi, 2005), making it the most concentrated amino acid in brain. The exact functional role of NAA in neurons and its significance of high concentrations have not been known. However, evidences indicate that NAA is an important precursor of the neuropeptide, N-acetylaspartylglutamate (NAAG) (Moffett et al., 2007). Other proposed cellular roles of NAA include osmoregulation of neuronal cells and axon-glial signaling, maintenance of nitrogen balance in brain, basic source of acetate required for myelin lipid biosynthesis in oligodendrocytes, and facilitation of energy metabolism in neuronal mitochondria etc. (Taylor et al., 1995; Clark, 1998; Baslow, 2003; Baslow et al., 2003; Madhavarao et al., 2005; Moffett et al., 2007). NAA is also a significant brain specific biomarker (Rael et al., 2004). The reduction in NAA (obtained from magnetic resonance spectroscopy, MRS of NAA) level has been used as an index of the extent of brain injury following a traumatic injury (Danielsen and Ross, 1999). Almost all neurological disorders involving neuronal loss or dysfunction (e.g., including amyotrophic lateral sclerosis, alzheimers, multiple sclerosis, epilepsy, schizophrenia, cerebral ischemia, and glioblastoma) are associated with alterations in NAA levels (Paley et al., 1996; Danielsen and Ross, 1999; Kantarci and Jack, 2003; Kalra and Arnold, 2004; Briellmann et al., 2005; Abbott and Bustillo, 2006; Criste and Trapp, 2006). Altered level of NAA is also associated with two inborn error of metabolism namely, Canavan disease (CD), in which there is a build-up of NAA (hyperacetylaspartia) and spongiform leukodystrophy (hypoacetylaspartia), caused due to the lack of aspartoacylase activity, where the enzyme that synthesizes NAA is apparently absent (Burlina et al., 1994; Boltshauser et al., 2004).

It has been known from various studies that NAA is an osmolyte in the brain cells responsible for the removal of water from neurons (Taylor et al., 1994, 1995). Most recently, our laboratory also reported that NAA exhibit strong stabilizing effect on different proteins (due to increase in *T*_m_ and $\Delta G_{\text{D}}{\;}^{\circ }$) (Warepam et al., 2020). Furthermore as mentioned above, there is alteration in the NAA levels under various neurodegenerative conditions. Since various neurodegenerative conditions are associated with toxic protein inclusions (which are in fact, aggregates, high order oligomers or amyloidogenic species), we thought, NAA could be a modulator of protein aggregation or proteopathic conditions. To address this issue, we have initiated our study on two different proteins (carbonic anhydrase, CA, and catalase), whose folding and aggregation pathways are well-known. We discovered that NAA is a potential inhibitor of protein aggregation. The results indicate that cellular overexpression or treatment of affected brain region with NAA can prevent brain cells from deposition of toxic protein inclusions.

## Materials and Methods

### Materials

Commercially lyophilized CA (catalog number C2624) and catalase (C9322) were purchased from Sigma. NAA was also obtained from the Sigma. Urea was purchased from MP Biomedicals. These chemicals were used without further purification as they are of analytical grade.

### Methods

#### Preparation of Protein Stock Solutions and Determination of Concentrations

CA and catalase solutions were dialyzed extensively against 0.1 M KCl at pH 7.0 and 4°C. The dialyzed protein stock solutions were filtered using 0.22 μm millipore syringe filter. A single protein band during polyacrylamide gel electrophoresis was obtained for both the proteins. Concentration of carbonic anhydrase and catalase solutions were determined experimentally using molar absorption coefficient, ε (M^−1^ cm^−1^) value of 57,000 at 280 nm for CA (Yazdanparast et al., 2005) and 3.24 × 10^5^ at 405 nm for catalase (Haber et al., 1993). For optical measurements all the proteins and buffer solutions were prepared in degassed double distilled water containing 0.1 M KCl. pH of the protein solutions remained similar even after addition of NAA.

#### Protein Aggregation Studies for CA and Catalase

CA (0.1 mg/ml in 0.05 M potassium phosphate buffer pH 7.0) was incubated overnight in presence of various concentrations of NAA at room temperature. Catalase (0.2 mg/ml) was also incubated with NAA for 1 h. Then, aggregation profiles were monitored by measuring the change in the light scattering intensity (at 500 nm) at 63°C for 15 min using Jasco V-660 UV/Vis spectrophotometer equipped with a Peltier type controller (ETCS-761) (for CA) and at 55°C for 70 min using Cary Eclipse Fluorescence Spectrophotometer (equipped with a single cell peltier) (for catalase). The measurements were carried out for at least three times. Kinetic parameters (lag time, *k*_app_ and *I*_f_) of CA and catalase aggregation were analyzed using the equation:

(1) $$\begin{array}{l} I=I_{o}+\;\frac{I_{f}}{1+e^{-\left(\frac{t-t_{o}}{b}\right)}} \end{array}$$

where *I* is the light scattering intensity at time *t*, and *t*_o_ is the time at 50% maximal light scattering, *I*_o_ and *I*_*f*_ represent the initial base line and final plateau line, respectively, *b* is a constant. Thus, the apparent rate constant, *k*_app_ for the formation of aggregates is given by 1/*b* and the lag time is given by *t*_o_-2*b* (Nielsen et al., 2001). Each curve was independently analyzed for the respective kinetic parameters and mean was calculated. The deviations (standard error) from the mean was then analyzed.

#### Transmission Electron Microscopy (TEM) of Carbonic Anhydrase

Protein samples incubated at 63°C in the presence and absence of NAA (1 M) were pelleted. Pelleted samples (10 μl) were placed on a copper grid and were air dried for 5–10 min. For negative staining of the samples, 1% of uranyl acetate solution was added on to the copper grid and again allowed to air dried. Samples were then examined under FEI Tecnai G2-200 kV HRTA transmission electron microscopy operating at 200 kV.

#### Temperature-Dependent Aggregation Studies

Temperature dependent aggregation of CA was monitored by measuring the change in the light scattering intensity at 500 nm using Jasco V-660 UV/Vis spectrophotometer equipped with a Peltier type controller (ETCS-761) with a heating rate of 1°C/min. This scan rate was found to provide adequate time for equilibration. Each sample was heated from 20 to 80°C. The measurements were repeated for three times. The temperature dependent aggregation curve of carbonic anhydrase was analyzed using the following empirical equation (Senisterra et al., 2006, 2010):

(2) $$\begin{array}{l} I=\;\frac{I_{f}}{1+\;e^{\left[\frac{T_{agg}-T}{b}\right]}} \end{array}$$

where *I* is the light scattering intensity at temperature *T, I*_*f*_ is the limiting value of light scattering at higher temperatures. *T*_*agg*_ is the temperature corresponding to the middle point of the transition, i.e., a temperature at which *I* = *I*_*f*_/2 and *b* is constant. Each curve was independently analyzed for the respective aggregation parameters and mean was calculated. The deviations (standard error) from the mean was then analyzed.

#### Urea-Induced Denaturation Measurements

Urea-induced denaturation of CA (0.125 μM) in presence and absence of NAA were followed by measuring changes at 355 nm as a function of urea concentration using Perkin Elmer-LS 55. Protein solutions at each urea concentration were kept for 6 h to allow equilibration. The optical transition data were converted into Δ*G*_D_, Gibbs energy change using the equation:

(3) $$\begin{array}{l} \Delta G_{\text{D}}=-RTln\left\{\left(y-y_{N}\right)/\left(y_{D}-y\right)\right\} \end{array}$$

where *R* is the universal gas constant, *T* is the temperature in Kelvin, *y* is the observed optical property and *y*_*N*_ and *y*_*D*_ are, respectively, the optical properties of the native and denatured protein molecules under the same experimental condition in which *y* has been determined. We then use linear extrapolation method for the analysis of $\Delta G_{\text{D}}{\;}^{\circ }$ (Pace, 1975; Santoro and Bolen, 1988), using the following equation:

(4) $$\Delta G_{\text{D}}=\Delta G_{\text{D}}{\;}^{\circ }-m_{d}[\text{GdmCl}],$$

where $\Delta G_{\text{D}}{\;}^{\circ }$ is the value of Δ*G*_D_ at 0 M denaturant, and *m*_d_ gives the linear dependence of Δ*G*_D_ on the [Urea]. Each curve was independently analyzed for the respective thermodynamic parameters and mean was calculated. The deviations (standard error) from the mean was then analyzed.

### Structural Measurements

Near-UV CD spectra of native state of CA were measured in the presence and absence of NAA at least three times in a J-810 (Jasco spectropolarimeter) equipped with a Peltier-type temperature controller (Jasco PTC-424S). Necessary subtraction for the contribution of blank from each spectrum of protein was performed. Protein concentration used was 0.5 mg/ml. The path length of the cuvette used was 10 mm. Routinely calibration of the CD instrument was done with D-10-camphorsulfonic acid.

Intrinsic fluorescence of carbonic anhydrase was measured in a Perkin Elmer-LS 55 at least for three times in absence and presence of NAA. Protein concentration used was 3 μM. The excitation wavelength was 297 nm. Necessary blank subtraction was made.

AC (0.1 mg/ml) was titrated with the desired concentrations of NAA and incubated overnight at room temperature. ANS (16-fold molar excess over CA concentration) is added on each CA samples and then incubated at 63°C for 15 min for equilibration. The fluorescence spectra of CA aggregates formed in the presence and absence of NAA were recorded from 380 to 600 nm with an excitation wavelength of 345 nm on a Perkin Elmer-LS 55 (Fluorescence spectrometer) using a quartz cuvette of 5 mm path length. All necessary background corrections were made. The measurements were carried out for at least three times.

### Reversal of Protein Aggregation

Aggregation of CA was carried out at 63°C using a heating block (Indogenix) for 15 min in the absence of NAA. Light scattering intensity (at 500 nm) at 63°C was measured immediately after addition of NAA using UV-visible spectrophotometer. All necessary subtraction of blank solutions was made.

## Results

### NAA Affects the Aggregation Kinetics of CA and Catalase

**Figure 1:**
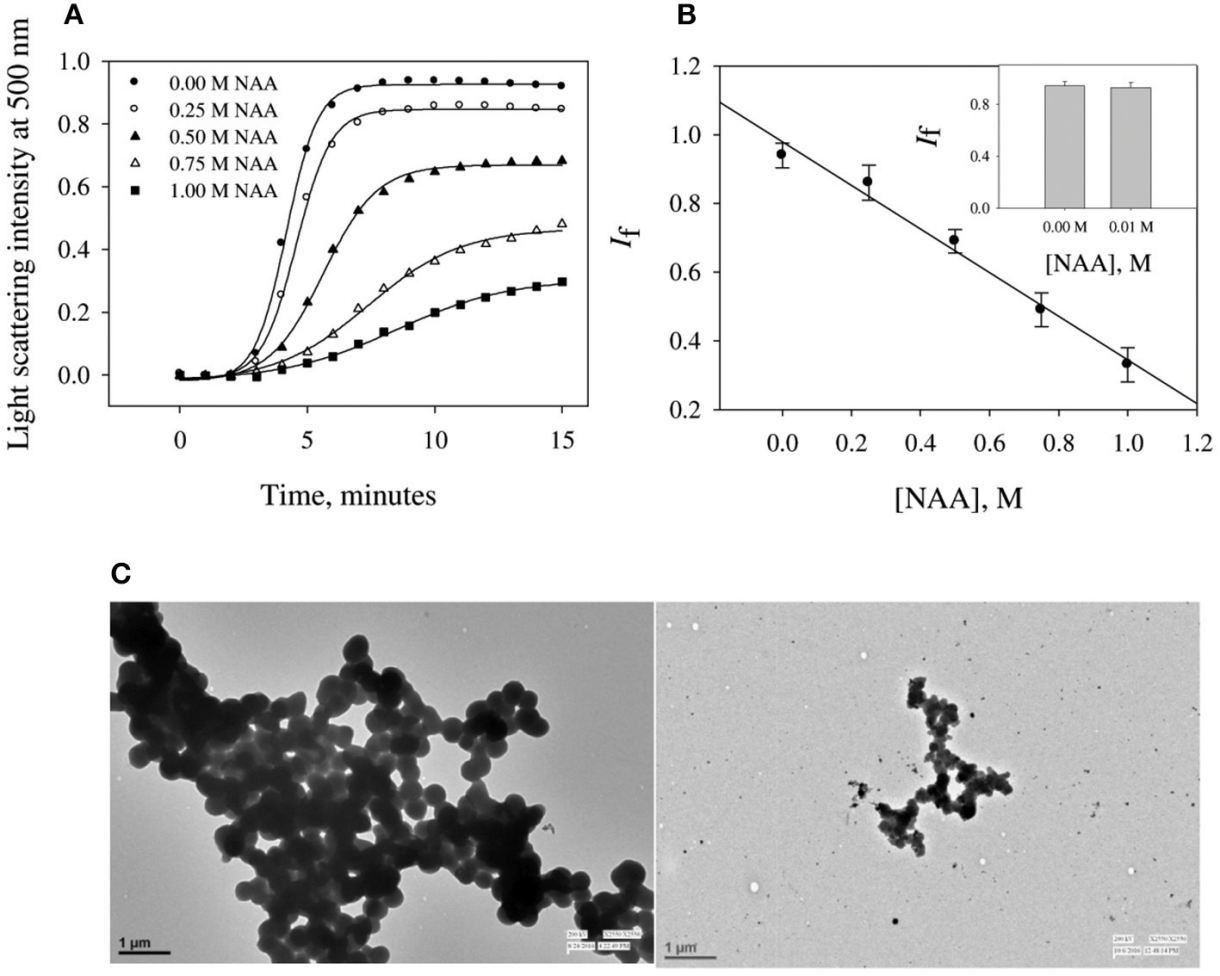
Time dependent aggregation profiles of carbonic anhydrase**. (A)** Aggregation kinetics of CA monitored by observing changes in the light scattering intensity at 500 nm in presence and absence of NAA. The lines present the best fits of the data using Equation (1). Results shown are representative of at least three independent measurements and mean error of the best fits are given in Table 1. **(B)** Plots of *I*_*f*_ vs. NAA concentrations. Error bars represents the standard errors of the mean. **(C)** Transmission electron microscopy images of CA in absence of NAA (left panel) and in presence (right panel) of 1 M NAA.

To investigate the effect of NAA on protein aggregation process, we have chosen CA as its folding behavior and aggregation kinetics have been extensively studied (Kundu and Guptasarma, 2002; Rana et al., 2008). A high content of β-sheet and a single domain structure protein make it highly prone to aggregate at an unfolding temperature of 63°C (Lavecchia and Zugaro, 1991; Sarraf et al., 2004). To study the effect of NAA on the aggregation of CA, we monitored the kinetic processes of CA aggregation at 63°C and pH 7.0 in the presence and absence of different concentrations of NAA by observing changes in the light scattering intensity at 500 nm. Figure 1A shows aggregation profile of CA as a function of time in absence and presence of different concentration of NAA. It is seen in this figure that there is decrease in the final magnitude of the aggregates suggesting its inhibitory effect. The aggregation kinetic parameters (lag time, *k*_app_ and *I*_*f*_) of CA in the absence and presence of different NAA concentrations were analyzed using Equation (1). The estimated values are given in Table 1. Results presented in this table show (i) an increase in lag phase in the presence of NAA and (ii) slower aggregation growth rates than those of the protein alone (control) and (iii) decrease in the *I*_*f*_ (final magnitude of aggregates formed). To further confirm the decrease in the extent of CA aggregation in presence of NAA, we have taken TEM images of CA aggregates in the presence of 1 M NAA concentration (Figure 1C). The large difference observed in the density of aggregates formed in the presence of NAA and that of the control (absence of NAA) suggest that NAA is able to decrease protein aggregation to a large extent.

**Table 1:** Kinetic parameters of CA aggregation in the absence and presence of NAA.

	**Concentration (M)**	**Lag time (min)**	***K*_app(min^−1^)_**	***I*_**f**_**
NAA	0.00	2.9 ± 0.16	1.63 ± 0.09	0.94 ± 0.05
	0.25	3.2 ± 0.17	1.46 ± 0.08	0.86 ± 0.05
	0.50	3.5 ± 0.17	0.95 ± 0.06	0.69 ± 0.05
	0.75	3.7 ± 0.19	0.55 ± 0.04	0.49 ± 0.04
	1.00	4.1 ± 0.24	0.45 ± 0.03	0.33 ± 0.03

*“±” represents standard error of three independent measurements*.

**Figure 2:**
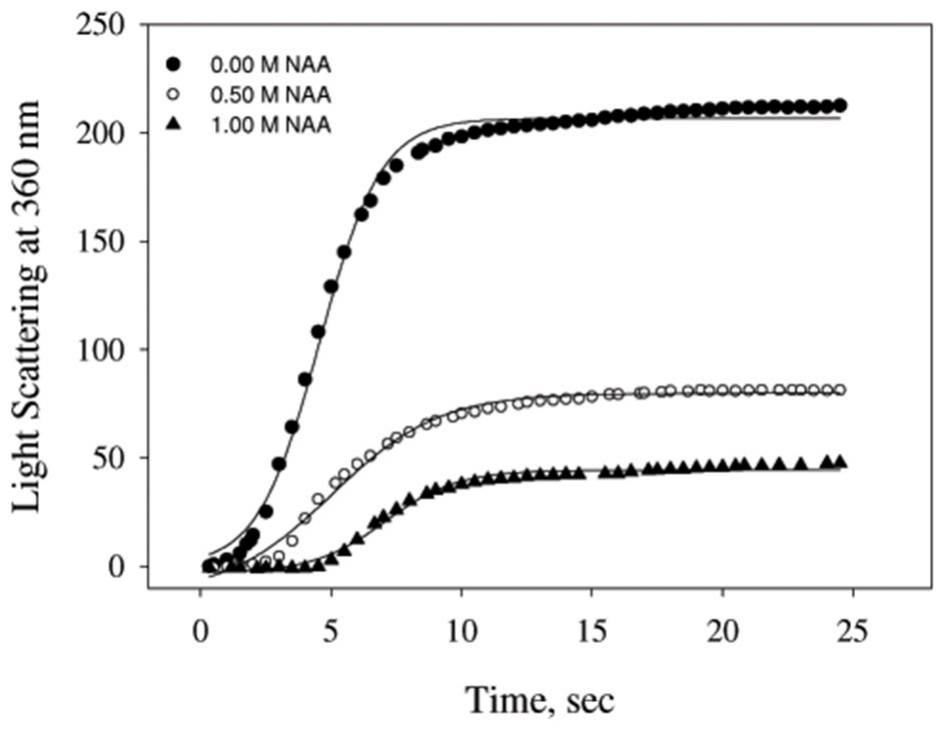
Time dependent aggregation profiles of catalase. Aggregation kinetics of catalase monitored by observing changes in the light scattering intensity at 360 nm in presence and absence of NAA. The lines present the best fits of the data using Equation (1). Results shown are representative of at least three independent measurements and mean error of the three independent best fits are given in Table 2.

We have also examined the ability of NAA on inhibiting the aggregation of another protein, catalase (Figure 2). The aggregation kinetic profiles were analyzed using equation (1) for the aggregation parameters (shown in Table 2). It is seen in this Table that NAA suppresses the aggregation of catalase and the behavior of inhibitory mechanism is similar to that observed for CA. The results indicate that NAA inhibits protein aggregation in general via common mechanism.

**Table 2:** Kinetic parameters of catalase aggregation in the absence and presence of NAA.

	**Concentration (M)**	**Lag time (min)**	**$k_{\text{app}({\text{min}}^{-1})}$**	***I_**f**_***
NAA	0.00	2.4 ± 0.14	0.9 ± 0.04	206.33 ± 14
	0.50	3.7 ± 0.19	0.9 ± 0.05	81.28 ± 7
	1.00	4.5 ± 0.22	0.8 ± 0.05	45.86 ± 4

“*±” represents standard error of three independent measurements*.

### NAA Increases the $\Delta G_{\text{D}}{\;}^{\circ }$ and *T_agg_* of CA

**Figure 3:**
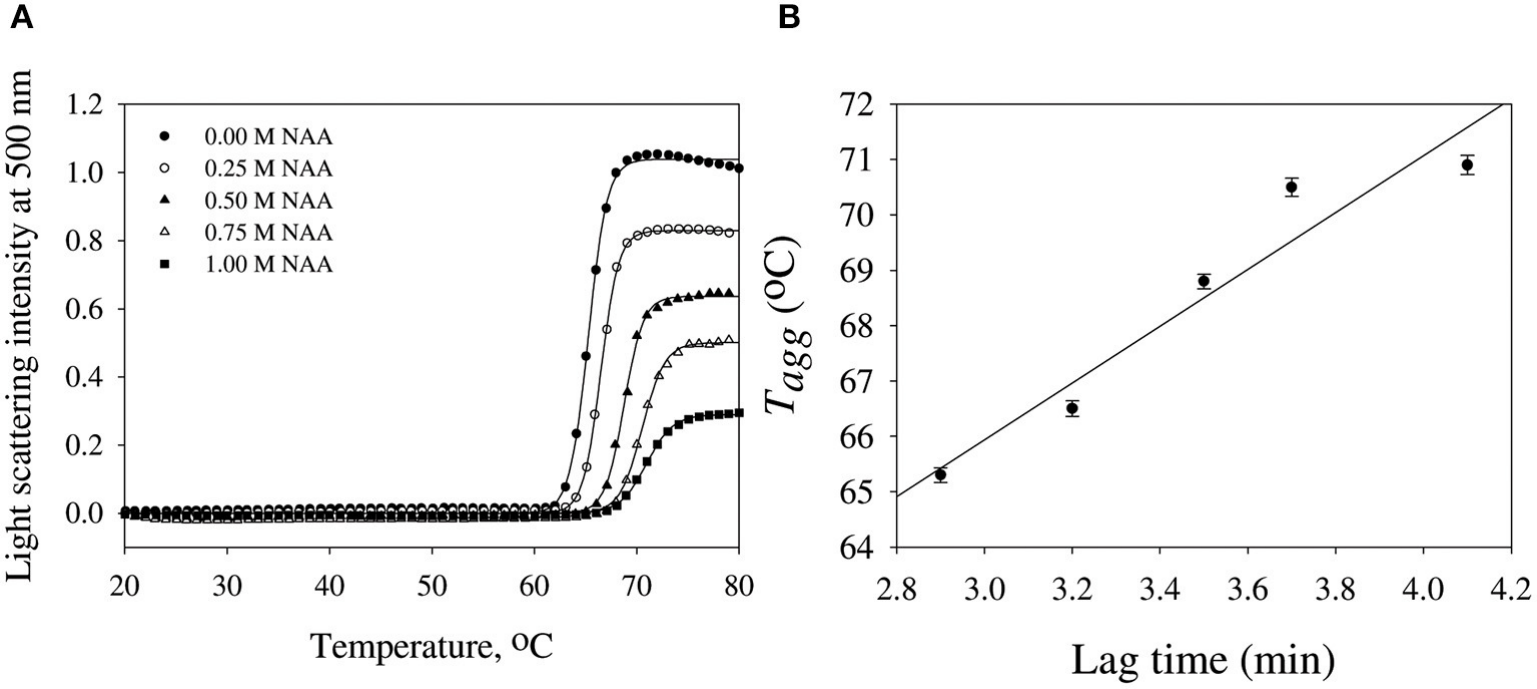
Temperature dependent aggregation profiles of carbonic anhydrase. **(A)** Temperature dependent aggregation kinetics of CA monitored by observing changes in the light scattering intensity at 500 nm in presence and absence of NAA. The lines present the best fits of the data using Equation (2). Results shown are representative of at least three independent measurements and mean error of the three independent best fits are given in Table 3. **(B)** Plots of *T*_agg_ vs. lag time.

**Figure 4:**
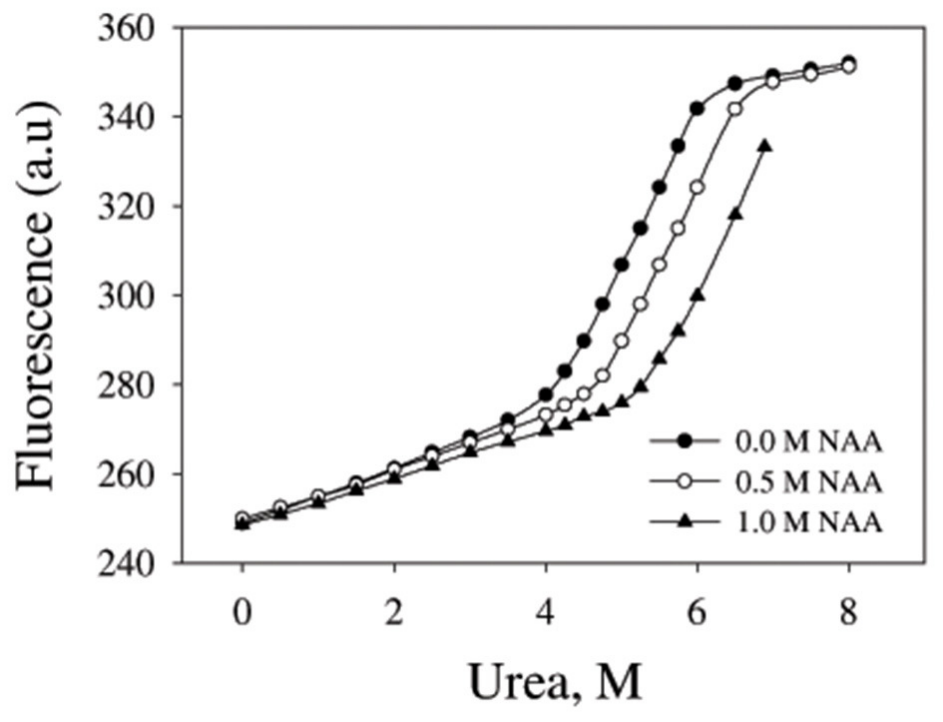
Urea denaturation profiles of CA. Denaturation of CA by observing changes at 355 nm using different concentration of urea in presence and absence of NAA. Excitation wavelength was 280 nm. Results shown are representative of at least three independent measurements and mean error of the three independent best fits are given in Table 4.

We have further investigated the effect of NAA on temperature-dependent aggregation of CA. Figure 3A shows the aggregation of CA as a function of temperature at pH 7.0 in the presence and absence of various concentrations of NAA. It is seen in the figure that CA initiates aggregation when it reaches 63°C in absence of NAA which is consistent with the earlier reports (Lavecchia and Zugaro, 1991; Sarraf et al., 2004). However, in the presence of NAA the aggregation starts at higher temperature. The parameters, *T*_agg_ and *I*_f_ of temperature-dependent aggregation of CA were further analyzed using appropriate equations (Equation 2). The evaluated values are given in Table 3. It is seen in the table that NAA is able to decrease CA aggregation by decreasing the *I*_f_ with increasing NAA concentration indicating that the extent of formation of aggregates is reduced. Accordingly, we also observed an increase in the *T*_agg_ value as a function of NAA concentration indicating stabilizing of the native protein by NAA. To directly estimate the changes in thermodynamic parameters, we have performed urea-induced denaturation of CA in presence of NAA (Figure 4) and the evaluated parameters (using Equations 3 and 4) are shown in Table 4. However, it should be noted that at 1 M NAA, a complete urea-induced transition curve could not be obtained due to experimental constraints and therefore *y*_D_ was unknown. Since *y*_D_ is independent of the molar concentration of NAA, we have analyzed this incomplete transition curve by using property of *y*_D_ for the control (in the absence of NAA). It is seen in Table 4 that there is increase in the *C*_m_ and $\Delta G_{\text{D}}{\;}^{\circ }$ of the CA in presence of NAA in a concentration dependent manner. There is around 25% increases in the $\Delta G_{\text{D}}{\;}^{\circ }$ in presence of 1 M NAA.

**Table 3:** Parameters of temperature dependent aggregation of CA in the absence and presence of NAA.

	**Concentration (M)**	***T_**agg**_* (°C)**	***I_**f**_***
NAA	0.00	65.3 ± 0.26	1.00 ± 0.06
	0.25	66.5 ± 0.22	0.83 ± 0.04
	0.50	68.8 ± 0.23	0.64 ± 0.04
	1.00	70.5 ± 0.18	0.50 ± 0.03
	1.50	70.9 ± 0.21	0.29 ± 0.02
	2.00	71.4 ± 0.24	0.24 ± 0.02

“*±” represents standard error of three independent measurements*.

**Table 4:** Thermodynamic stability parameter of carbonic anhydrase in presence of NAA.

**[NAA], M**	***C*_**m**_ (M)**	**$\Delta G_{\text{D}}{\;}^{\circ }$ (kcal/mol)**
0.0	5.2 ± 0.06	7.9 ± 0.39
0.5	5.7 ± 0.05	8.6 ± 0.43
1.0	6.3 ± 0.08	9.9 ± 0.47

“*±” represents standard error of three independent measurements*.

### NAA Induces Partial Structural Alteration on Native CA

**Figure 5:**
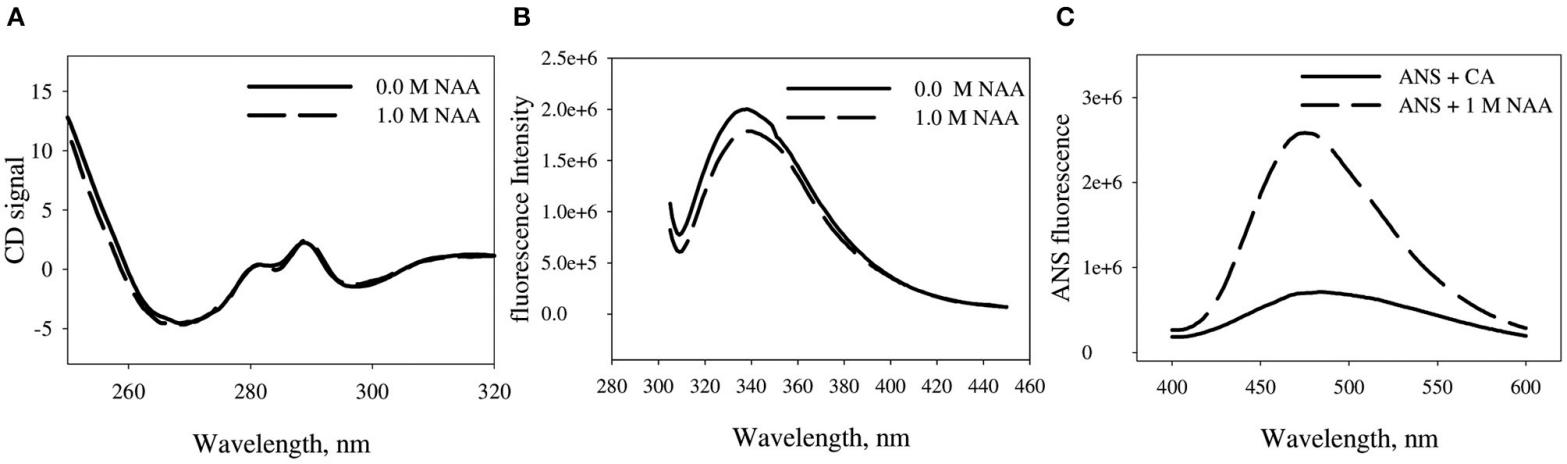
Effect of NAA on native state conformation of CA. **(A)** Near UV-CD spectra (tertiary structure) of CA in absence and presence of NAA **(B)** Intrinsic fluorescence emission spectra of CA in absence and presence of NAA **(C)** ANS emission fluorescence spectra of CA. The spectra shown are representative of three independent measurements.

We have further investigated the effect of NAA on the structural differences between the NAA treated and untreated CA. Figure 5 shows the near-UV CD, tryptophan fluorescence and ANS fluorescence spectra of the CA in absence and presence of CA. Far-UV CD spectra could not be collected due to experimental constraints. It is seen in the Figure that CA exhibit three different peaks in the near-UV region (270, 287, and 296 nm) which are characteristics peaks originated from the trp47, trp97, and trp245, respectively. There is no observed alteration in any of the peaks in presence of NAA. However, tryptophan spectra shows little decrease in the λ_max_ of the CA indicating that some of the tryptophan residues are partly in a polar environment relative to the NAA untreated samples. ANS measurement revealed that native CA does not bind ANS but the NAA treated CA exhibit partial binding of ANS.

### NAA Partially Reverses Preformed CA Aggregates

**Figure 6:**
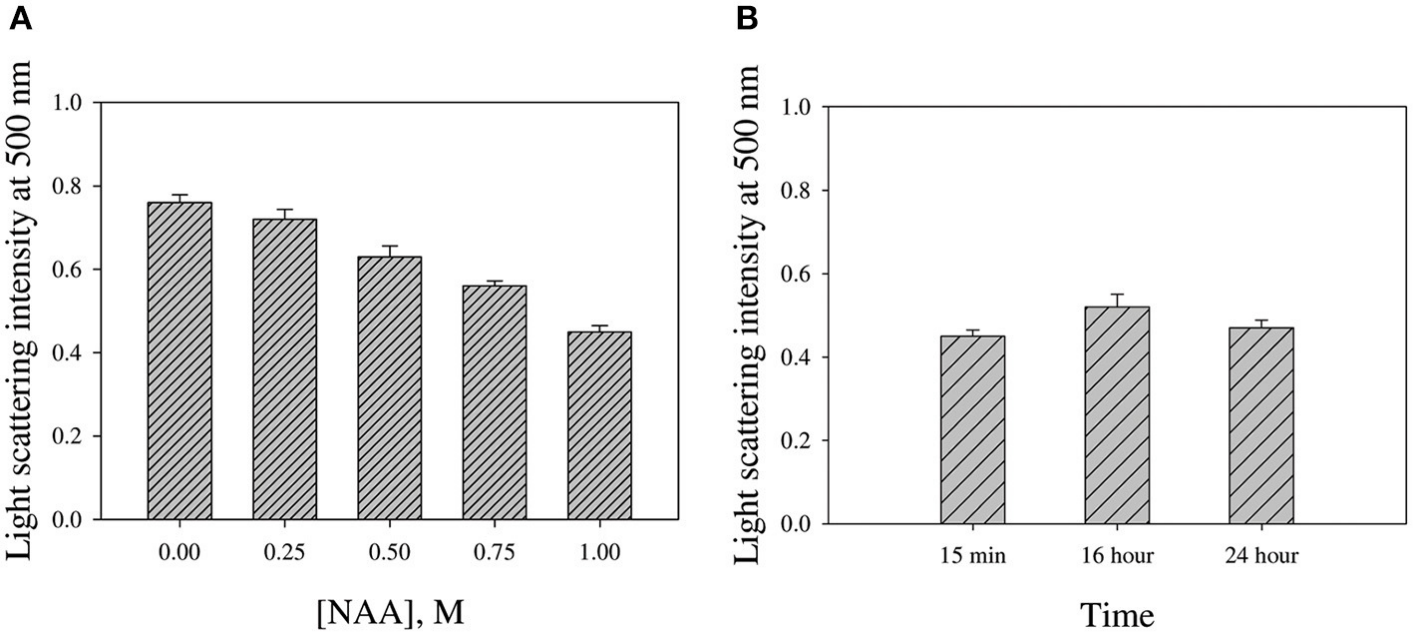
NAA effect on reversing preformed CA aggregates. **(A)** Light scattering intensities of aggregated CA in the presence of different NAA concentrations **(B)** Intensities upon prolong incubations in presence of 1.0 M NAA. Error bars represent standard error of the mean.

We were interested to investigate if NAA could reverse the preformed CA aggregates. For this we monitored the change in light scattering intensity (at 500 nm) of the various CA aggregated samples diluted in buffer alone and in presence of NAA. Figure 6 shows the plot of light scattering intensity vs. NAA concentrations. It is seen in the figure that NAA is able to partly reverse the preformed CA aggregates.

## Discussion

NAA is not only the most abundant amino acid in brain cells (as compared to the other amino acids) but also seems to play an important role in the proteopathic conditions as its level is altered under disease conditions associated with the proteopathies (Moffett et al., 2007). Our results revealed that NAA exhibits pronounce inhibitory effect on the aggregation behavior of CA (see Figures 1A,B) as evident from the decrease in final magnitude of the aggregates in a concentration dependent manner. However, physiological concentration of NAA could not offer any inhibitory effect (see inset Figure 1B). TEM images further confirmed that CA forms spherical aggregates and there is several fold decrease in amount of aggregates formed (Figure 1C). The observed inhibitory effects on catalase aggregation (Figure 2) also indicate that aggregation suppressing effect of NAA is not confined to CA but may be generally true. It has been well-understood that deposition of Aβ or alpha-synuclein oligomers is the common pathology associated with Alzheimer or Parkinson, respectively (Rabbani and Choi, 2018). Our results indicate that in the presence of NAA, Aβ or alpha-synuclein aggregation should be prevented. In support, it has been reported that NAA suppresses the aggregation of Aβ42 (Dollé et al., 2018). Thus, it appears that increase in the Aβ or alpha synuclein aggregation under the pathologic conditions, is due to decrease in the NAA concentration making it insufficient to modulate aggregation pathway. Furthermore, increase in intracellular level of myo-inositol under the neurodegenerative conditions might also be an attempt to augment the aggregation of Aβ or alpha synuclein. It is likely that cells undergo genetic and epigenetic alteration due to the pathological conditions that is in favor of aggregate formation.

We then investigated the mechanism of suppression of CA aggregation by NAA. For this first of all, NAA-induced aggregation profiles were analyzed for its effect on the various steps of the oligomerization process (as shown in Table 1). In general protein aggregation profile is exemplified by a sigmoidal curve consisting of lag phase (or nucleation step), log phase (oligomerization step), and final stationary phase (or formation of mature aggregates). The general belief is that native protein exists in dynamic equilibrium with aggregation prone species (APS) formed by undergoing certain conformational changes in the native protein. Nucleation phase comprises of the conversion of the native protein to the APS. The APS then self-associates to form various high order oligomers in the log phase. High order oligomers further assemble in an ordered (producing amyloidogenic species) or disordered (producing amorphous oligomers) manner to form mature aggregated species (maturation step). It is also evident in table that inhibitory effect of NAA on the CA aggregation pathway is largely due to its effect on the nucleation step (as evident from the pronounce increase in the lag time), which subsequently reduces the oligomerization step (*k*_app_). Since native and APS exists in a dynamic equilibrium, the observed effect of the nucleation step perhaps, be due to stabilization of the native state thereby shifting the equilibrium, Native ↔ APS toward the left. This hypothesis was established firstly by monitoring the temperature-dependence of CA aggregation process using light scattering as a probe (see Figure 3A and Table 3). Shifting of the temperature-dependent aggregation curve to higher temperatures (or *T*_agg_) in a NAA concentration-dependent manner is a signature for the stabilization of the native protein or reduction of the APS population (Mittal and Singh, 2014). To further confirm, we have plotted *T*_agg_ as a function of the lag phase (Figure 3B). A strong correlation between the two parameters confirms that the decrease in *I*_f_ is a result of the effect of NAA on the nucleation phase. A direct evidence came from the urea-induced unfolding studies of CA in the Figure 4. It is seen in Table 4 that $\Delta G_{\text{D}}{\;}^{\circ }$ of CA increases in presence of NAA suggesting the stabilization of CA native state by NAA. NAA has also been shown to increase the *T*_m_ and $\Delta G_{\text{D}}{\;}^{\circ }$ of lysozyme and RNase-A (Warepam et al., 2020). Taken together, the results led us to conclude that stabilization of the native state might have therefore disfavoured partial unfolding of CA and thus the formation of APS.

We were further interested to investigate the conformational alteration on CA due to NAA treatment that results in inhibition of CA aggregation. For this we have measured the conformation of CA in presence of NAA using multiple spectroscopic techniques. It is seen in Figure 5A that there is no significant alteration in the gross tertiary structure of the proteins due to NAA (as evident from near-UV CD studies). On the other hand, tryptophan fluorescence studies revealed that there is slight changes in the environment of the tryptophan (as evident from the slight hypochromicity) (Figure 5B). Such structural change affecting tryptophan micro-environment might have affected the exposition pattern of hydrophobic clusters to the solvent (Rabbani et al., 2012). ANS is a dye that specifically binds to the exposed hydrophobic clusters to the solvent (Rabbani et al., 2011, 2014, 2015). It is seen in Figure 5C that there no apparent binding of ANS in presence of NAA but significant binding with little shift in λ_max_ in absence indicating little compaction of the hydrophobic groups. Taken together, the results indicate that NAA treatment induces minute conformational alteration to the CA bringing about large increase in native state stability.

We were further interested to investigate if NAA could also dissolve the preformed aggregates. For this we have intentionally treated the pre-aggregated CA with different concentrations of NAA and analyzed the aggregation status using light scattering. It is evident in Figure 6A that there is partial decrease in the light scattering intensity in presence of NAA. However, there is no complete reversal of the aggregates even upon longer time incubations (Figure 6B). On molecular level, it is possible that the CH_3_ groups in NAA compete with the CH_3_-CH_3_ interaction between any two adhering polypeptides in the oligomer resulting in the disruption of the intermolecular hydrophobic interaction and hence masking off the exposed hydrophobic groups of the monomeric units upon dissociation from the oligomer. Taken together, we conclude that NAA not only suppresses protein aggregation but also partly dissolve preformed aggregates. Thus, our study clearly indicates that NAA is not only a protein stabilizer but also a potent inhibitor of protein aggregation. The inhibition of protein aggregation is via its effect on the nucleation step of the aggregation kinetics. Under pathological conditions of Alzheimers and Parkinson, NAA is highly lowered. Thus, attempt to increase intracellular NAA concentration would be a viable strategy toward the therapeutic intervention of amyloidogenic condition of brain. Nevertheless, NAA may also be employed in other cell types wherein deposition of protein inclusion is the main cause of the pathophysiology.

Similar to our observation on NAA, other protein chaperones including yeast HSP104 and human HSP40/HSP70/HSP110 or Fas apoptosis inhibitory molecule also have the ability to solubilize preformed aggregates *in vivo* in ATP dependent or ATP independent manners respectively (Shorter, 2011; Kaku et al., 2020). As compared to the other protein chaperones which work by their catalytic ability, NAA is a small molecule metabolite that perhaps bring about solubilization of oligomers by virtue of its ability to bind to the hydrophobic groups exposed in the protein aggregates. The observations indicate there might be existence of different disaggregases in the cellular environment.

## Data Availability Statement

The original contributions presented in the study are included in the article/supplementary material, further inquiries can be directed to the corresponding author/s.

## Author Contributions

LS was responsible for the conception, design, interpretation of the results, and writing manuscript. MW for conducting experiments. LS, MW, and AM involved in data analysis. GS, KK, SK, MK, and HR was involved in editing the final manuscript. AM and MK were involved in critically revising and editing the final manuscript. All authors contributed to the article and approved the submitted version.

## Conflict of Interest

The authors declare that the research was conducted in the absence of any commercial or financial relationships that could be construed as a potential conflict of interest.

## References

[B1] Abbott C. Bustillo J. (2006). What have we learned from proton magnetic resonance spectroscopy about schizophrenia? A critical update. Curr. Opin. Psychiatry 19, 135–139. 10.1097/01.yco.0000214337.29378.cd 16612192

[B2] Baslow M. H. (2003). Brain N-acetylaspartate as a molecular water pump and its role in the etiology of Canavan disease: a mechanistic explanation. J. Mol. Neurosci. 21, 185–190. 10.1385/JMN:21:3:185 14645985

[B3] Baslow M. H. Suckow R. F. Gaynor K. Bhakoo K. K. Marks N. Saito M.. (2003). Brain damage results in down-regulation of N-acetylaspartate as a neuronal osmolyte. Neuromol. Med. 3, 95–104. 10.1385/NMM:3:2:95 12728192

[B4] Birnbaum S. M. Levintow L. Kingsley R. B. Greenstein J. P. (1952). Specificity of amino acid acylases. J. Biol. Chem. 194, 455–470. 10.1016/S0021-9258(18)55898-1 14927637

[B5] Blüml S. (1999). *In vivo* quantitation of cerebral metabolite concentrations using natural abundance 13C MRS at 1.5 T. J. Magn. Reson. 136, 219–225. 10.1006/jmre.1998.1618 9986765

[B6] Boltshauser E. Schmitt B. Wevers R. A. Engelke U. Burlina A. B. Burlina A. P. (2004). Follow-up of a child with hypoacetylaspartia. Neuropediatrics 35, 255–258. 10.1055/s-2004-821036 15328569

[B7] Briellmann R. S. Wellard R. M. Jackson G. D. (2005). Seizure-associated abnormalities in epilepsy: evidence from MR imaging. Epilepsia 46, 760–766. 10.1111/j.1528-1167.2005.47604.x 15857444

[B8] Burlina A. P. Corazza A. Ferrari V. Erhard P. Künnecke B. Seelig J.. (1994). Detection of increased urinary N-acetylaspartylglutamate in Canavan disease. Eur. J. Pediatr. 153, 538–539. 10.1007/BF01957015 7957376

[B9] Clark J. B. (1998). N-acetyl aspartate: a marker for neuronal loss or mitochondrial dysfunction. Dev. Neurosci. 20, 271–276. 10.1159/000017321 9778562

[B10] Criste G. Trapp B. (2006). N-acetyl-L-aspartate in multiple sclerosis. Adv. Exp. Med. Biol. 576, 199–214. 10.1007/0-387-30172-0_14 16802714

[B11] Danielsen E. Ross B. (1999). Magnetic Resonance Spectroscopy Diagnosis of Neurological Diseases. New York, NY: Marcel Dekker.

[B12] Dollé J. P. Rodgers J. M. Browne K. D. Troxler T. Gai F. Smith D. H. (2018). Newfound effect of N-acetylaspartate in preventing and reversing aggregation of amyloid-beta *in vitro*. Neurobiol. Dis. 117, 161–169. 10.1016/j.nbd.2018.05.023 29859874 PMC6553457

[B13] Goldstein F. B. (1959). Biosynthesis of N-acetyl-L-aspartic acid. J.Biol.Chem. 234, 2702–2706. 10.1016/S0021-9258(18)69763-713670942

[B14] Haber J. Maślakiewicz P. Rodakiewicz-Nowak J. Walde P. (1993). Activity and spectroscopic properties of bovine liver catalase in sodium bis(2-ethylhexyl)sulfosuccinate/isooctane reverse micelles. Eur. J. Biochem. 217, 567–573. 10.1111/j.1432-1033.1993.tb18278.x 7693463

[B15] Kaku H. Ludlow A. V. Gutknecht M. F. Rothstein T. L. (2020). FAIM opposes aggregation of mutant SOD1 that typifies some forms of familial amyotrophic lateral sclerosis. Front. Neurosci. 14:110. 10.3389/fnins.2020.00110 32153351 PMC7047752

[B16] Kalra S. Arnold D. L. (2004). ALS surrogate markers MRS Amyotroph Lateral Scler Other. Motor Neuron Disord. 5, 111–114. 10.1080/17434470410019861 15512889

[B17] Kantarci K. Jack C. R. Jr. (2003). Neuroimaging in Alzheimer disease: an evidence-based review. Neuroimaging Clin. N. Am. 13, 197–209. 10.1016/S1052-5149(03)00025-X 13677801

[B18] Knizley H. Jr. (1967). The enzymatic synthesis of N-acetyl-L-aspartic acid by a water insoluble preparation of a cat brain acetone powder. J. Biol. Chem. 242, 4619–4622. 10.1016/S0021-9258(18)99502-5 6061408

[B19] Kundu B. Guptasarma P. (2002). Use of a hydrophobic dye to indirectly probe the structural organization and conformational plasticity of molecules in amorphous aggregates of carbonic anhydrase. Biochem. Biophys. Res. Commun. 293, 572–577. 10.1016/S0006-291X(02)00257-7 12054640

[B20] Lavecchia R. Zugaro M. (1991). Thermal denaturation of erythrocyte carbonic anhydrase. FEBS Lett. 292, 162–164. 10.1016/0014-5793(91)80858-Z 1959599

[B21] Madhavarao C. N. Arun P. Moffett J. R. Szucs S. Surendran S. Matalon R.. (2005). Defective N-acetylaspartate catabolism reduces brain acetate levels and myelin lipid synthesis in Canavan's disease. Proc. Natl. Acad. Sci. U. S. A. 102, 5221–5226. 10.1073/pnas.0409184102 15784740 PMC555036

[B22] Mittal S. Singh L. R. (2014). Macromolecular crowding decelerates aggregation of a β-rich protein, bovine carbonic anhydrase: a case study. J. Biochem. 156, 273–282. 10.1093/jb/mvu039 24917682

[B23] Miyake M. Kakimoto Y. Sorimachi M. (1981). A gas chromatographic method for the determination of N-acetyl-L-aspartic acid, N-acetyl-aspartylglutamic acid and beta-citryl-L-glutamic acid and their distributions in the brain and other organs of various species of animals. J. Neurochem. 36, 804–810. 10.1111/j.1471-4159.1981.tb01665.x 7205274

[B24] Moffett J. R. Ross B. Arun P. Madhavarao C. N. Namboodiri A. M. (2007). N-Acetylaspartate in the CNS: from neurodiagnostics to neurobiology. Prog Neurobiol. 81, 89–131. 10.1016/j.pneurobio.2006.12.003 17275978 PMC1919520

[B25] Nielsen L. Khurana R. Coats A. Frokjaer S. Brange J. Vyas S.. (2001). Effect of environmental factors on the kinetics of insulin fibril formation: elucidation of the molecular mechanism. Biochemistry 40, 6036–6046. 10.1021/bi002555c 11352739

[B26] Pace C. N. (1975). The stability of globular proteins. CRC Crit. Rev. Biochem. 3, 1–43. 10.3109/10409237509102551 238787

[B27] Paley M. Cozzone P. J. Alonso J. Vion-Dury J. Confort-Gouny S. Wilkinson I. D.. (1996). A multicenter proton magnetic resonance spectroscopy study of neurological complications of AIDS. AIDS Res. Hum. Retroviruses 12, 213–222. 10.1089/aid.1996.12.213 8835199

[B28] Pan J. W. Takahashi K. (2005). Interdependence of N-acetyl aspartate and high-energy phosphates in healthy human brain. Ann. Neurol. 57, 92–97. 10.1002/ana.20317 15546136

[B29] Rabbani G. Ahmad E. Vahid Khan M. Ashraf M. T. Bhat R. Khan R. H. (2015). Impact of structural stability of cold adapted Candida antarctica lipase B (caLB): in relation to pH, chemical and thermal denaturation. RSC Adv. 5, 20115–20131. 10.1039/C4RA17093H

[B30] Rabbani G. Ahmad E. Zaidi N. Fatima S. Khan R. H. (2012). pH-induced molten globule state of rhizopus niveus lipase is more resistant against thermal and chemical denaturation than its native state. Cell Biochem. Biophys. 62, 487–499. 10.1007/s12013-011-9335-9 22215307

[B31] Rabbani G. Ahmad E. Zaidi N. Khan R. H. (2011). pH-dependent conformational transitions in conalbumin (Ovotransferrin), a metalloproteinase from hen egg white. Cell Biochem. Biophys. 61, 551–560. 10.1007/s12013-011-9237-x 21833676

[B32] Rabbani G. Choi I. (2018). Roles of osmolytes in protein folding and aggregation in cells and their biotechnological applications. Int. J. Biol. Macromol. 109, 483–491. 10.1016/j.ijbiomac.2017.12.100 29274422

[B33] Rabbani G. Kaur J. Ahmad E. Khan R. H. Jain S. K. (2014). Structural characteristics of thermostable immunogenic outer membrane protein from *Salmonella enterica* serovar Typhi. Appl. Biochem. Biotechnol. 98, 2533–2543. 10.1007/s00253-013-5123-3 23949993 PMC7080034

[B34] Rael L. T. Thomas G. W. Bar-Or R. Craun M. L. Bar-Or D. (2004). An anti-inflammatory role for N-acetly aspartate in stimulated human astroglial cells. Biochem. Biophys. Res. Commun. 319, 847–853. 10.1016/j.bbrc.2004.04.200 15184060

[B35] Rana A. Gupta T. P. Bansal S. Kundu B. (2008). Formation of amyloid fibrils by bovine carbonic anhydrase. Biochim. Biophys. Acta 1784, 930–935. 10.1016/j.bbapap.2008.02.020 18395531

[B36] Santoro M. M. Bolen D. W. (1988). Unfolding free energy changes determined by the linear extrapolation method. 1. Unfolding of phenylmethanesulfonyl alpha-chymotrypsin using different denaturants. Biochemistry. 27, 8063–8068. 10.1021/bi00421a014 3233195

[B37] Sarraf N. S. Saboury A. A. Ranjbar B. Moosavi-Movahedi A. A. (2004). Structural and functional changes of bovine carbonic anhydrase as a consequence of temperature. Acta Biochim. Pol. 51, 665–671. 10.18388/abp.2004_3551 15448728

[B38] Senisterra G. A. Ghanei H. Khutoreskaya G. Dobrovetsky E. Edwards A. M. Privé G. G.. (2010). Assessing the stability of membrane proteins to detect ligand binding using differential static light scattering. J. Biomol. Screen. 15, 314–320. 10.1177/1087057109357117 20150591

[B39] Senisterra G. A. Markin E. Yamazaki K. Hui R. Vedadi M. Awrey D. E. (2006). Screening for ligands using a generic and high-throughput light-scattering-based assay. J. Biomol. Screen. 11, 940–948. 10.1177/1087057106294699 17092916

[B40] Shorter J. (2011). The mammalian disaggregase machinery:Hsp110 synergizes with Hsp70 and Hsp40 to catalyse protein disaggregation and reactivation in a cell-free system. PLoS One. 6:e26319 10.1371/journal.pone.0026319 22022600 PMC3194798

[B41] Taylor D. L. Davies S. E. Obrenovitch T. P. Doheny M. H. Patsalos P. N. Clark J. B.. (1995). Investigation into the role of N-acetylaspartate in cerebral osmoregulation. J. Neurochem. 65, 275–281. 10.1046/j.1471-4159.1995.65010275.x 7790871

[B42] Taylor D. L. Davies S. E. Obrenovitch T. P. Urenjak J. Richards D. A. Clark J. B.. (1994). Extracellular Nacetylaspartate in the rat brain: *in vivo* determination of basal levels and changes evoked by high K+. J. Neurochem. 62, 2349–2355. 10.1046/j.1471-4159.1994.62062349.x 8189239

[B43] Truckenmiller M. E. Namboodiri M. A. Brownstein M. J. Neale J. H. (1985). N-Acetylation of L-aspartate in the nervous system: differential distribution of a specific enzyme. J.Neurochem. 45, 1658–1662. 10.1111/j.1471-4159.1985.tb07240.x 4045470

[B44] Warepam M. Ahmad K. Rahman S. Rahaman H. Kumari K. Singh L. R. (2020). N-Acetylaspartate is an important brain osmolyte. Biomolecules 10, 286. 10.3390/biom10020286 32059525 PMC7072545

[B45] Yazdanparast R. Khodarahmi R. Soori E. (2005). Comparative studies of the artificial chaperone-assisted refolding of thermally denatured bovine carbonic anhydrase using different capturing ionic detergents and beta-cyclodextrin. Arch. Biochem. Biophys. 437, 178–185. 10.1016/j.abb.2005.03.003 15850557

